# Modern active surveillance in low- and intermediate-risk prostate cancer without re-biopsy

**DOI:** 10.1038/s41391-025-01010-6

**Published:** 2025-09-14

**Authors:** Jale Lakes, Rouvier Al-Monajjed, Isabelle Busshoff, Anne Hübner, Matthias Boschheidgen, Birte Valentin, Gerald Antoch, Peter Albers, Lars Schimmöller, Jan Philipp Radtke

**Affiliations:** 1 Department of Urology, Medical Faculty, University of Düsseldorf, Düsseldorf, Germany; 2 Division of Personalized Early Detection of Prostate Cancer, German Cancer Research Center (DKFZ), Heidelberg, Germany; 3 Department of Diagnostic and Interventional Radiology, Medical Faculty, University of Düsseldorf, Düsseldorf, Germany; 4 Center for Integrated Oncology Düsseldorf (CIO Aachen, Bonn, Cologne, Düsseldorf), CIO, Düsseldorf, Germany; 5 Department of Diagnostic, Interventional Radiology and Nuclear Medicine, Marien Hospital Herne, University Hospital of the Ruhr-University Bochum, Herne, Germany; 6 Department of Radiology, German Cancer Research Center (DKFZ), Heidelberg, Germany

**Keywords:** Outcomes research, Cancer therapy

## Introduction

Active surveillance (AS) has become a widely accepted management strategy for men with low-risk localized prostate cancer (PC), aiming to minimize overtreatment and associated morbidities [1–3]. Its application in intermediate-risk PC, however, remains a subject of ongoing debate. This perspective article examines the current evidence, selection criteria, and outcomes associated with AS in both low- and intermediate-risk PC populations with particular focus on the role of MRI to reduce follow-up biopsies.

## Active surveillance in low-risk PC

For men with low-risk PC, AS is the preferred standard management approach and recommended in recent guidelines [1–3]. Studies have demonstrated that many of these tumours are indolent, with a low likelihood of progression. The primary advantage of AS in this group is the avoidance of immediate radical treatments, thereby preserving quality of life without compromising cancer-specific survival [1, 4]. Long-term data indicate that men on AS have a low incidence of metastasis and prostate cancer-specific mortality [1, 5]. For example, a large prospective study of 1818 patients with very low-risk or low-risk PC showed a cumulative PC-specific mortality rate of 0.1% at both 10 and 15 years after AS [6]. These long-term data imply that AS is appropriate and oncologically safe for patients with low-risk disease. However, it should be noted that within the framework of the relatively intensive structured AS program in recent studies, ~20–50% of men had to undergo active treatment within the first 5–10 years [6–8].

In general, patients whose initial diagnostics included MRI/TRUS-fusion prostate biopsy had a lower risk of reclassification during AS and, consequently, a reduced need for active treatment. This appears to reinforce the role of multiparametric MRI (mpMRI) in the selection of suitable candidates for AS [1, 7, 9].

Due to the heterogeneous selection criteria of AS and the lack of prospective randomized studies, the guideline group of the European Association of Urology (EAU) has initiated the so-called DETECTIVE study as a structured project to standardize AS and evaluate existing protocols [10].

In addition to the clinical criteria of ISUP GG 1, clinical tumour stage cT1c or cT2a, and a PSA value of <10 ng/mL, PSA density <0.15 ng/mL/cc was also proposed as a selection criterion based on systematic biopsy (SB) schemes [10]. However, the DETECTIVE panel also recommended to expand AS to selected intermediate-risk PC cases.

## Expanding AS to intermediate-risk prostate cancer

Intermediate-risk PC encompasses a heterogeneous group, typically histologically defined by ISUP grade group (GG) 2 or 3, or clinical stage T2b-T2c. The suitability of AS for this cohort is less clear due to the increased potential for disease progression compared to low-risk patients. Recent studies have explored the feasibility of AS in selected intermediate-risk patients [7, 11]. The rationale for including these patients into AS programs is comparable results on biochemical recurrence, metastases-free survival, and cumulative mortality in ISUP GG 1 and 2 PC in some studies [12–14]. In particular, in the recent 15-year analysis of the PROTECT trial, 25% of those men who were allocated to active monitoring as a treatment option had intermediate-risk, still having comparable results in metastases-free and cancer specific-survival [12]. A meta-analysis by Baboudjian et al. assessed treatment-free and oncologic outcomes in intermediate-risk patients managed with AS [11]. The study reported that intermediate-risk patients had similar treatment-free survival to low-risk patients but faced significantly higher risks of metastasis and PC mortality. Notably, these results often comprise ISUP GG 2 and 3 patients, and in a subgroup analysis focusing on patients with ISUP GG 2, treatment-free and metastasis-free survival rates were comparable to those of low-risk patients [11]. This finding underscores the importance of careful patient selection within the intermediate-risk category, particularly favouring those with low-volume ISUP GG 2 disease [11]. The decision to pursue AS in intermediate-risk patients necessitates stringent selection criteria to identify those most likely to benefit [15]. Factors favouring AS include ISUP GG2 with minimal Gleason pattern 4 involvement, low tumour volume, and absence of adverse pathological features such as intraductal carcinoma or cribriform patterns [10, 11, 15]. Advanced imaging techniques, particularly mpMRI, play a crucial role in assessing tumour characteristics and guiding biopsy strategies, with comparable results as in low-risk PC patients on AS [7]. In addition, PSMA PET-CT may lead to a better selection of patients suitable for AS and is currently being tested in the CONFIRM trial [16].

Monitoring protocols for patients on AS typically involve regular PSA testing, digital rectal examinations, periodic imaging, and repeat biopsies to detect any signs of disease progression promptly [2, 3]. The frequency and intensity of monitoring may be adjusted based on individual risk factors and initial tumour characteristics, particularly in favourable intermediate-risk PC. This has been implemented recently by different risk groups, including histopathologic risk by Cambridge Prognostic Groups, PSA density, and MRI, the so-called STRATCANS risk groups [17, 18]. In example, there was a very low risk of AS disqualification in men with a STRATCANS 1 (out of 3), defined by CPG 1 and a prestine MRI [18]. Thus, a follow-up MRI could safely be scheduled 5 years after AS initiation, whereas a STRATCAN 3 needed annual MRI scans [18]. Standardized criteria to review serial MRI scans during AS foster to decrease the number of follow-up biopsies in AS protocols, thus aim to increase AS acceptance [7, 19–21]. Most recently, de Vos et al., demonstrated in a dataset of 2512 patients from the PRIAS trial that a dynamic model incorporating clinical parameters (age, PSA, prostate volume, previous negative biopsy, PSA velocity) and MRI can predict AS disqualification and if a cut-off of 15% to be disqualified is used, two-thirds of follow-up biopsies can be avoided and only 4.6% of disqualifications are missed. Interestingly, the GAP3 database served as validation cohort [22, 23].

## The role of MRI in AS protocols

Recent evidence and guidelines suggest that MRI and MRI-guided biopsies are crucial before AS is considered [3, 24]. An upfront MRI and an MRI-guided biopsy led to a reduction in AS disqualification rate from 59% to 20% in a 4-year follow-up period [9].

The necessity of a combined MRI fusion TB and SB is clearly demonstrated by the aforementioned meta-analysis [24]. In combination, 27% of patients with a higher tumour grade are detected in the re-biopsy, compared to 17% or 20% with TB or SB alone, respectively [24]. If a PI-RADS 3–5 lesion is present on the MRI, the detection of a higher tumour grade with the combined approach increases to 35%, compared to 24% for each of the individual methods [24]. The positive impact of a combination of TB and SB on the detection rate of higher tumour grades under AS was also shown by Klotz et al. in a prospective randomized study [25].

In the near future, MRI probably will replace repeat biopsies in AS strategies. However, the use of MRI in this context needs quality-controlled MRIs.

This initial lack of standardization in the reporting of MRI for determining disease progression on AS led to the development of recommendations for the application of prostate MRI in the AS setting. Such recommendations were introduced as the Prostate Cancer Radiological Estimation of Change in Sequential Evaluation (PRECISE) system [19].

**Fig. 1:**
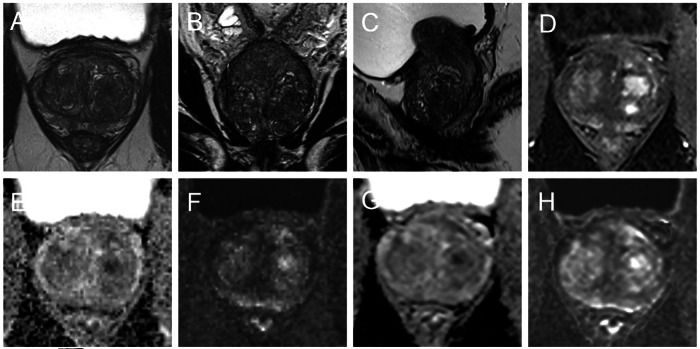
68 age, Status post positive TRUS biopsy (1/12 cores; left mid anterior; max. infiltration 40%; ISUP GG1; T1c, G2). PSA stable 6.4 ng/ml. Prostate volume 79 ml, 78 ml on prior MRI. PSA density 0.08 ng/ml/ml, stable 0.08 ng/ml/ml. Intravesical Prostate protusion (IPP): 23 mm. Follow-up MRI (**A**-**H**) Atypical hyperplasia at the apex and left anterior, no relevant progress. PI-RADS (v2.1): 3 PRECISE-Score (v2): 3-nonV MR Staging: cT2a.

**Fig. 2:**
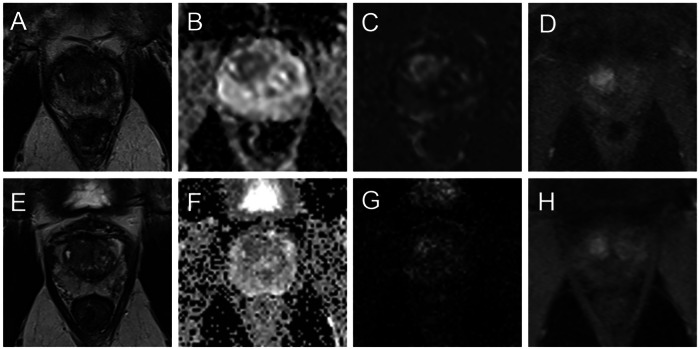
72 age, Status post MRI/TRUS-fusion biopsy (05/2021; 0/12 systematic cores; 2/6 targeted cores, TZa apex right side/5a; max. infiltration 90%; Gleason score 3 + 3 = 6, ISUP GG1). Rising PSA-level (PSA recently 15 ng/ml; previously 04/2021 PSA 12.7 ng/ml). Prostate volume 41 ml, constant 41 ml (04/2021). PSA density 0.37 ng/ml/ml, previously 0.30 ng/ml/ml (04/2021). Comparison of recent MRI **A**–**D** and first MRI (**E**–**H**). Progression of the anterior lesion. PI-RADS (v2.1): 5. PRECISE-Score (v2): 4 MR Staging: cT2c Follow-up biopsy: ISUP GG 2 (80% core infiltration) Radical prostatectomy: pT2c pN0 (0/13) L0 V0 Pn0 R0 ISUP GG 2.

PRECISE provides a standardized approach to evaluate for progression on follow-up MRI in patients on AS, seeking to avoid ambiguity in the reporting of MRI examinations in this setting [19]. PRECISE uses a 1-to-5 Likert scale to score the likelihood of progression on MRI [19]. The determination of a specific score is based on changes in lesion size and/or conspicuity [19]. Importantly, such changes across follow-up examinations are evaluated in comparison with the patient’s baseline MRI (please see Figs. 1–2 for details) [19].

Recently, the second version of the PRECISE consensus recommendations for reporting of MRI in AS has been developed [20]. The novel recommendations include information on scan quality using Prostate Imaging-Quality (PI-QUAL) scores, to help to ensure a reliable framework for MRI quality and reporting [26]. However, the recent GLIMPSE study demonstrates variation in MR image quality even in experienced centers with room for improvement, in particular in dynamic contrast-enhanced imaging. Thus, following basic principles of the Prostate Imaging Reporting and Data System technical recommendations and training, as well as feedback from expert radiologists, is needed to improve widespread quality optimization [27]. Second, the PRECISE v2 panel determined that a 50% change in volume should be considered significant for any given lesion, as should an increase in PI-RADS score or T stage [20]. However, this was based on consensus rather than any specific evidence [20]. Additionally, various methods exist for determining lesion size, and the panel did not reach a consensus on how specific imaging planes should be used for measurement. Third, the panel agreed that for fair assessment of the change in size over time, the reading radiologist should remeasure lesions on both the baseline and the most recent prior scans. In the case of multiple lesions, a PRECISE score should be read for each lesion, and the score for the overall scan should be taken as the worst score.

Besides this, unlike PI-RADS score, the PRECISE score is lacking reference to any pathologic gold standard, such as a change in grade, and the implementation of the case report form as a structured template in electronic health record systems seems challenging [28].

On the other hand, PRECISE scoring has been widely adopted with promising results. Dias et al. summarized in a systematic review from eight prospective studies using PRECISE v1, a sensitivity between 59 and 100% for determining a histopathologic progression, whereas ruling out of AS disqualification was possible with a negative predictive value (NPV) of 70–96% [29]. Interestingly, the PRECISE criteria have also been expanded to AS in intermediate-risk PC, with a comparable sensitivity of 78–90% and NPV of 90% [7, 30]. These results are a promising requisite to reduce the number of follow-up biopsies by structured MRI strategies on AS [7, 21].

## Conclusion

AS remains a cornerstone in the management of low-risk and selected intermediate-risk PC, with favourable features such as low-volume ISUP GG two disease. MRI has become an integral part of inclusion to AS programs and in the follow-up. Specifically, the potential to reduce follow-up biopsies, thus increasing patients‘ acceptance and individualized follow-up schedules, has been demonstrated by standardized MRI scoring systems with high negative predictive values to rule out progression of disease [7, 21, 22, 30, 31]. Ongoing research on reclassification and disqualification patterns and recommendations for patients is essential to refine selection criteria. This is of particular interest, as there is a wide heterogeneity on thresholds to stop AS, and the most reliable, Gleason Score reclassification, has been demonstrated to occur in approximately only 13% and 14% of cases in a recent systematic review and results of the GAP3 consortium [32, 33]. Further research will comprise genomic testing and, more easily, the influence of different percentages of Gleason pattern 4, and long-term data to refine selection criteria further and answer the fundamental question, how clinical and MRI parameters should be integrated to optimize management strategies for this diverse patient population [34].
